# Quick outpatient diagnosis in small district or general tertiary hospitals: A comparative observational study

**DOI:** 10.1097/MD.0000000000006886

**Published:** 2017-06-02

**Authors:** Elisabet Montori-Palacín, Sergio Prieto-González, Ignasi Carrasco-Miserachs, Jordi Altes-Capella, Yaroslau Compta, Alfons López-Soto, Xavier Bosch

**Affiliations:** a Unit of Internal Medicine, Hospital Plató, University of Barcelona; b Department of Systemic Autoimmune Diseases, Hospital Clínic, University of Barcelona, Biomedical Research Institute August Pi i Sunyer (IDIBAPS); c Neurology Service, Hospital Clínic/Department of Biomedicine, University of Barcelona; d Department of Internal Medicine, Hospital Clínic, University of Barcelona, Institut d’Investigació Biomèdica August Pi i Sunyer (IDIBAPS), Barcelona, Catalonia, Spain.

**Keywords:** community hospitals, district hospitals, general tertiary hospitals, malignancy, outpatients, quick diagnosis units

## Abstract

While quick diagnosis units (QDUs) have expanded as an innovative cost-effective alternative to admission for workup, studies investigating how QDUs compare are lacking. This study aimed to comparatively describe the diagnostic performance of the QDU of an urban district hospital and the QDU of its reference general hospital.

This was an observational descriptive study of 336 consecutive outpatients aged ≥18 years referred to the QDU of a urban district hospital in Barcelona (QDU1) during 2009 to 2016 for evaluation of suspected severe conditions whose physical performance allowed them to travel from home to hospital and back for visits and examinations. For comparison purposes, 530 randomly selected outpatients aged ≥18 years referred to the QDU of the reference tertiary hospital (QDU2), also in Barcelona, were included. Clinical and QDU variables were analyzed and compared.

Mean age and sex were similar (61.97 (19.93) years and 55% of females in QDU1 vs 60.0 (18.81) years and 52% of females in QDU2; *P* values = .14 and .10, respectively). Primary care was the main referral source in QDU1 (69%) and the emergency department in QDU2 (59%). Predominant referral reasons in QDU1 and 2 were unintentional weight loss (UWL) (21 and 16%), anemia (14 and 21%), adenopathies and/or palpable masses (10 and 11%), and gastrointestinal symptoms (10 and 19%). Time-to-diagnosis was longer in QDU1 than 2 (12 [1–28] vs 8 [4–14] days; *P* < .001). Malignancy was more common in QDU2 than 1 (19 vs 13%; *P* = .001). Patients from both groups with malignancy, aged ≥65 years and requiring >2 visits to be diagnosed were in general more likely to be males, to have UWL and adenopathies and/or palpable masses but less likely anemia, to undergo more examinations except endoscopy, and to be referred onward to specialist outpatient clinics.

Despite some differences, results showed that, for diagnostic purposes, the overall performance and effectiveness of QDUs of urban district and reference general hospitals in evaluating patients with potentially serious conditions were similar. This study, the first to compare the performance of 2 hospital-based QDUs, adds evidence to the opportunity of producing standardized guidelines to optimize QDUs infrastructure, functioning, and efficiency.

## 1 Introduction

Despite the advantages of a universal organization, public health system has also their inherent risks. In Spain, unrestricted access has produced overuse and abuse, with a system responding to patient demands rather than needs.^[1,2]^ With the arrival of the financial crisis in 2008, many European countries with public health systems tried to face the challenge of their viability and sustainability with rather unpopular measures such as exclusion from public funding of some tests and treatments without added value and copayments.^[3–5]^ Improvements in efficiency have also been pursued through mergers of hospital centers, accelerating the switch from the traditional mode focused on hospitalization to outpatient care, and primary care interventions.^[6–8]^ It has been recommended to improve the quality of primary care through a better follow-up of chronic diseases such as chronic obstructive pulmonary disease, asthma and diabetes mellitus in order to avoid unnecessary inpatient admissions.^[9,10]^ Indeed, inappropriate admissions are frequently used as an indicator of problems of access to primary care.^[10]^ Outpatient alternatives have also been promoted to directly reducing the number of hospitalizations and lengths of stays both for treatment (e.g., day care hospitals, home hospitalization programs, telemedicine) and diagnostic purposes.^[8,10–14]^ While conventional hospitalizations for diagnostic workup are considered inappropriate admissions,^[8,15,16]^ they have been estimated to account for 15% to 20% of inpatients in internal medicine wards of Spanish hospitals.^[17,18]^ An innovative cost-effective alternative to this type of admissions was the creation of the so-called quick diagnosis units (QDUs).^[19]^

The first QDU was established in the United Kingdom, at the Queen Elizabeth Hospital in Birmingham, and was based on an outpatient diagnostic assessment of patients with clinical suspicion of malignancy, with guide-symptoms driving the appropriate specialist (e.g., breast masses—gynecologists).^[20]^ Although inspired by the Birmingham experience, QDUs are slightly different: they are led by general internists and are not exclusively centered on cancer, even though suspicion of cancer constitutes the main reason for referral to these units.^[19,21,22]^ Considering the reduction in the rate of admissions for workup and the corresponding hospitalization costs from switching to QDUs, the reported evidence relative to these units including data about referral criteria, organization, and effectiveness is very limited.^[22–27]^

While published studies are mostly from Spanish groups, the first QDU was created in 1996 in the community Hospital of Granollers. Its results were reported in 2004 in a Spanish medical journal.^[26]^ A subsequent study published in 2009 in an international medical journal about the experience of the Hospital of Granollers QDU and the general tertiary Hospital Clínic of Barcelona QDU revealed a similar diagnostic effectiveness, markedly lower costs, and greater patient satisfaction scores compared with traditional admission for the same evaluable disorders.^[19]^ Although numerous QDUs have emerged during the last 15 years in several hospitals including general tertiary centers such as the Hospital Clinic of Barcelona and the Hospital of Bellvitge and smaller community centers such as the Hospital of Granollers, no study has evaluated how QDUs compare between them. The purpose of this observational descriptive study was to characterize the diagnostic performance of the QDU of an urban district hospital in Barcelona in assessing patients with potentially serious disorders and compare it with the QDU of its reference general hospital.

## 2 Materials and methods

### 2.1 Settings

The QDU of Hospital Plató (QDU1) was created in 2009 and is integrated in the internal medicine department of this hospital. It is an urban district hospital in Barcelona providing care to a reference population of 140,000. It has 120 beds for acute patients and the general Hospital Clínic is its reference center. The unit evaluates patients with suspected severe conditions whose physical performance allows them to travel from home to hospital and back for visits and examinations. Similar to inpatients, diagnostic tests are preferentially arranged. The attending physician in charge of the unit is a general internist who dedicates 4 hours per week to this clinical duty. The QDU of Hospital Clínic (QDU2) was created in 2005 and is also integrated in the internal medicine department of this hospital and, in particular, in an adult day-care center. It is a public tertiary academic hospital in Barcelona with 855 beds and a reference population of almost 550,000. Like QDU1, QDU2 evaluates patients with potentially serious diseases with a preserved general status that allows them to undergo an outpatient scheduled study. QDU2 staff includes a consultant general internist full-time, a senior internal medicine resident, a nurse part-time, a nurse coordinator part-time, and 2 secretaries part-time. The unit is open 5 hours a day, 4 days a week.

### 2.2 Study design and population

We evaluated 336 consecutive patients aged ≥18 years referred to QDU1 between November 2009 and February 2016. For comparative purposes, we also retrospectively analyzed 530 patients aged ≥18 years who were referred to QDU2. These patients were chosen randomly, using a computer-generated numbers table, from 5250 consecutive patients who were assessed at QDU2 during the same period. All the patients were referred to the 2 units from primary care centers (PCCs), hospital emergency departments (EDs), outpatient clinics, and from inpatients wards (e.g., patients admitted to evaluate a certain condition who were prematurely discharged as they were deemed to be suitable for study on an outpatient basis). The main referral clinical criteria of QDU1 were the same as those of QDU2.^[19,24]^ Referrals were made by the hospital computerized information system, phone calls, and emails. All the patients were scheduled at least for the first visit. The patients lost before the first visit or before the evaluation concluded were excluded from the study.

The working protocol of the 2 units consists of a quick first appointment (usually within 5 days of referral) followed by preferential programming of diagnostics tests and subsequent visits until a diagnosis is made. For QDU1, there is an a priori expectation of not more than 15 days between the first and the last visit and of, ideally, up to 2 visits to make a diagnosis. Diagnostic examinations were conducted either at the corresponding hospitals or, when unavailable at Hospital Plató, at other centers (principally its reference hospital). The diagnosticians in QDU1 and 2 did not change throughout the study period.

The study received approval by the research ethics committee of the network of hospitals to which Hospital Plató belongs to (CEIC—Unió Catalana d’Hospitals) and by the research ethics committee of Hospital Clínic. All the patients from the QDU1 group provided written informed consent. Informed consent was waived for QDU2 patients, who were retrospectively evaluated.

### 2.3 Database

Data from all the patients evaluated were recorded onto case report forms and codified in a database. Recorded data included sex, age, number of patients aged ≥65 years, sources of referral, date of and clinical reason for referral, date of first QDU visit, time to first visit (interval between referral and first visit at QDU), number of visits, successive-to-first visit ratio, type and number of diagnostic examinations, waitimg time to examination (interval between QDU physician's order and the examination being actually performed), final diagnosis, time-to-diagnosis (interval between first visit at QDU and final diagnosis), number of patients needing ≤2 visits to achieve a diagnosis, and onward referrals.

### 2.4 Analysis of data

Qualitative variables were compared using the χ2 test or the Fisher exact test, as appropriate, and are expressed as absolute frequencies (%). Quantitative variables with a normal distribution were compared using the Student *t* test and are expressed as means with standard deviations (SD). The nonparametric Mann–Whitney *U* test was used, when appropriate, to compare continuous variables with skewed distributions and the results are expressed as medians with interquartile ranges [IQR]. Statistical significance was established at .05. Analyses were done with SPSS software (version 21.0) (SPSS, Chicago, IL).

## 3 Results

Table 1 shows the general characteristics of patients from the 2 groups including demographic data, referral sources, clinical reasons for referral, time to first visit, and QDU outcomes during the diagnostic evaluation including, among others, final diagnosis (malignancy/no malignancy) and onward referrals. Overall, 23 patients (12 QDU1 and 11 QDU2 patients) were lost to follow-up. Among QDU2 patients, there was a minimal rate of missing data on the variable “time to first visit” and complete data on other variables.

**Table 1:**
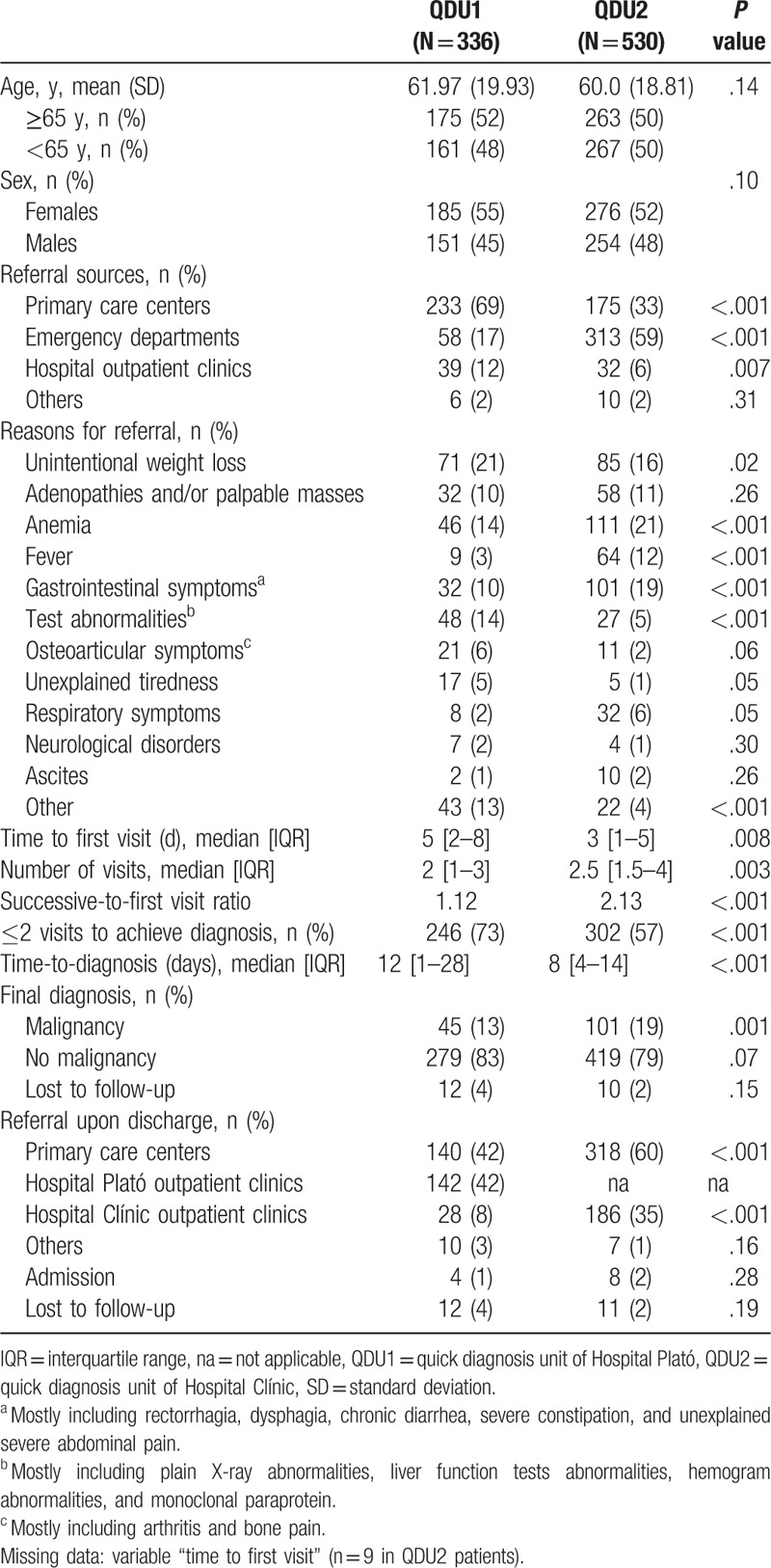
General characteristics of study patients.

Mean age and sex were not significantly different (61.97 (19.93) years and 55% of females in QDU1 vs 60.0 (18.81) years and 52% of females in QDU2; *P* values = .14 and .10, respectively). While QDU1 patients were predominantly referred from PCCs (69%), EDs were the main referral source of QDU2 subjects (59%). The most frequent reasons for referral to QDU1 were unintentional weight loss (UWL) (21%), tests detected at referral sites (herein referred to as test abnormalities (mostly laboratory and plain X-ray abnormalities)) (14%), anemia (14%), abnormal peripheral lymphadenopathy and/or palpable masses (10%), and gastrointestinal symptoms (10%). In QDU2, the most common referral reasons were anemia (21%), gastrointestinal symptoms (19%), UWL (16%), fever (12%), and adenopathies and/or palpable masses (11%). The median time to first visit was longer in QDU1 than in QDU2 patients (5 [2–8] vs 3 [1–5] days; *P* = .008) and the median number of visits was lower in QDU1 patients (2 [1–3] vs 2.5 [1.5–4], respectively; *P* = .003). The 336 first visits in QDU1 generated 375 successive visits with a successive-to-first visit ratio of 1.12, which was lower than the QDU2 ratio (2.13) (*P* < .001). While QDU1 patients were more likely than QDU2 to require ≤2 visits to achieve a diagnosis (73 vs 57%; *P* < .001), the median time-to-diagnosis was longer in the former (12 [1–28] vs 8 [4–14] days, respectively; *P* < .001). Although a final diagnosis of no malignancy prevailed over malignancy in both QDU1 and 2 patients (83 and 79%), malignancy was more common among the latter (19 vs 13%, respectively; *P* = .001). After diagnosis, most QDU1 patients were referred to outpatient clinics of Hospital Plató and PCCs (42% each), with 8% being referred to specialist outpatient clinics of the reference Hospital Clínic. In addition, 60% of QDU2 patients were referred onward to PCCs and 35% to the hospital outpatient clinics (Table 1).

Table 2 shows the comparative frequency of diagnostic examinations and waiting times to each examination in the 2 groups. At first visit, nearly all the patients underwent laboratory tests and plain X-rays (98 and 87%, respectively, in QDU1 and 97 and 90%, respectively, in QDU2). The QDU2 patients underwent significantly more ultrasonographies, endoscopies, and cytology/biopsy studies than the QDU1 patients. Furthermore, significant differences were observed in the waiting times to computed tomography (CT) scan and cytology/biopsy studies, which were longer in QDU2 patients, and in the waiting times to ultrasonography, endoscopy, scintigraphy, and body fluorodeoxyglucose-positron emission tomography (FDG-PET), which were longer in QDU1 patients.

**Table 2:**
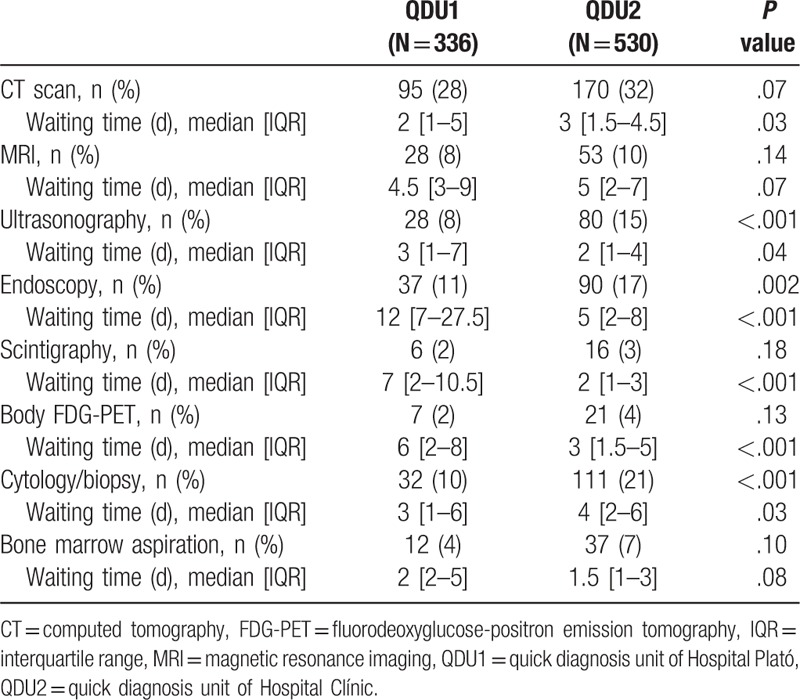
Main diagnostic examinations and waiting times.

Analyses according to patients’ age (<65 vs ≥65 years), number of visits needed to make a diagnosis (≤2 vs >2), and final diagnosis (malignancy vs no malignancy) in the 2 groups are detailed in Tables 3–5. The most relevant statistically significant results according to age are shown in Table 3. While QDU1 patients aged ≥65 years were less likely to have fever as a cause for referral than those aged <65 years, older QDU2 patients were more likely than younger patients to have UWL, adenopathies and/or palpable masses and gastrointestinal symptoms but less likely to have anemia. Moreover, QDU2 patients aged ≥65 years were more likely than those aged <65 years to be males, to undergo several examinations except endoscopy, which was more commonly performed, albeit not significantly, among younger patients, and to be referred onward to specialist outpatient clinics. When comparing the 2 QDU groups according to patients’ age, significant differences were observed in anemia and gastrointestinal symptoms as referral reasons, number of patients needing ≤2 visits, time-to-diagnosis, number of several examinations, and onward referrals (Table 3).

**Table 3:**
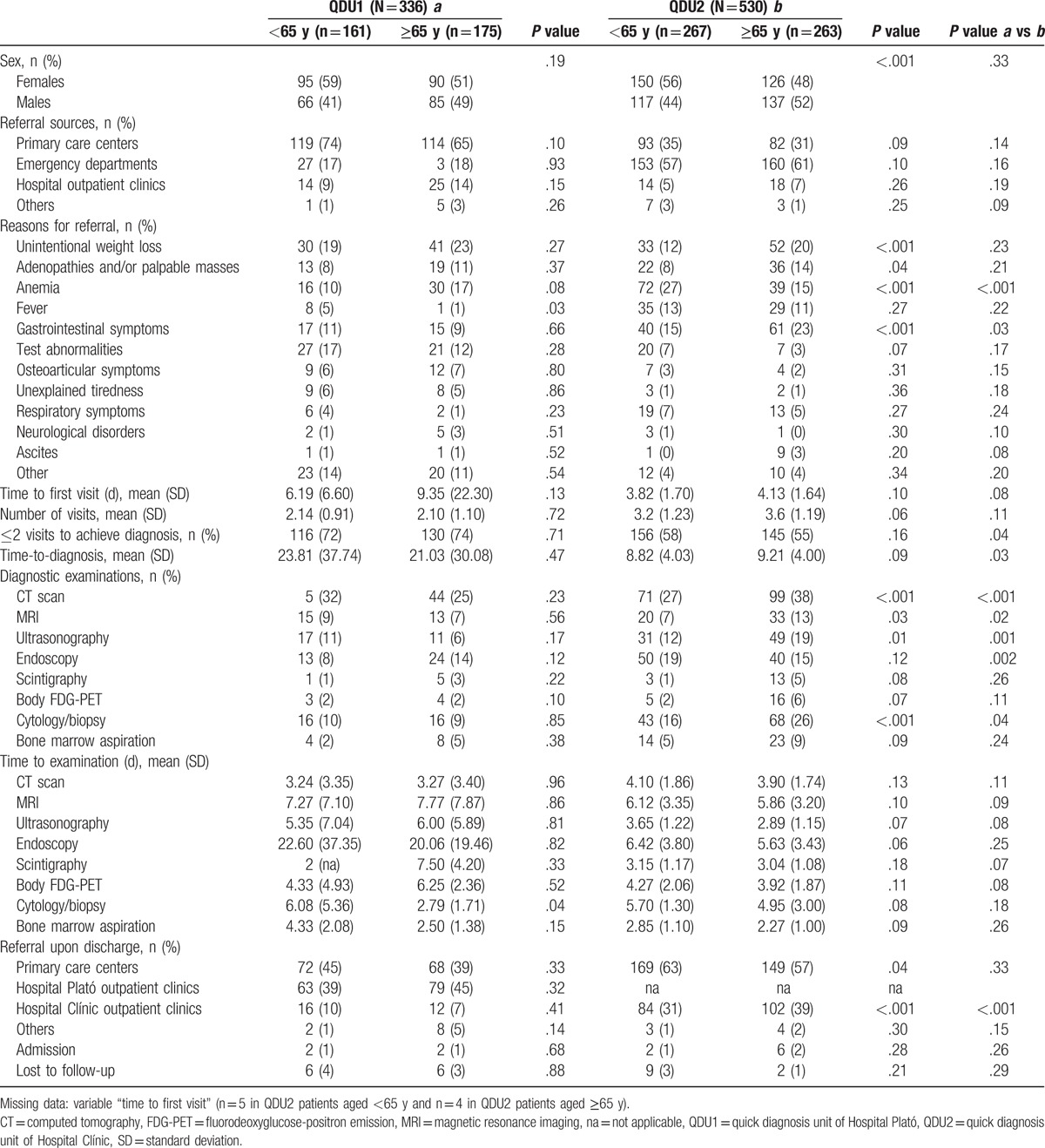
Comparative analyses according to age.

**Table 4:**
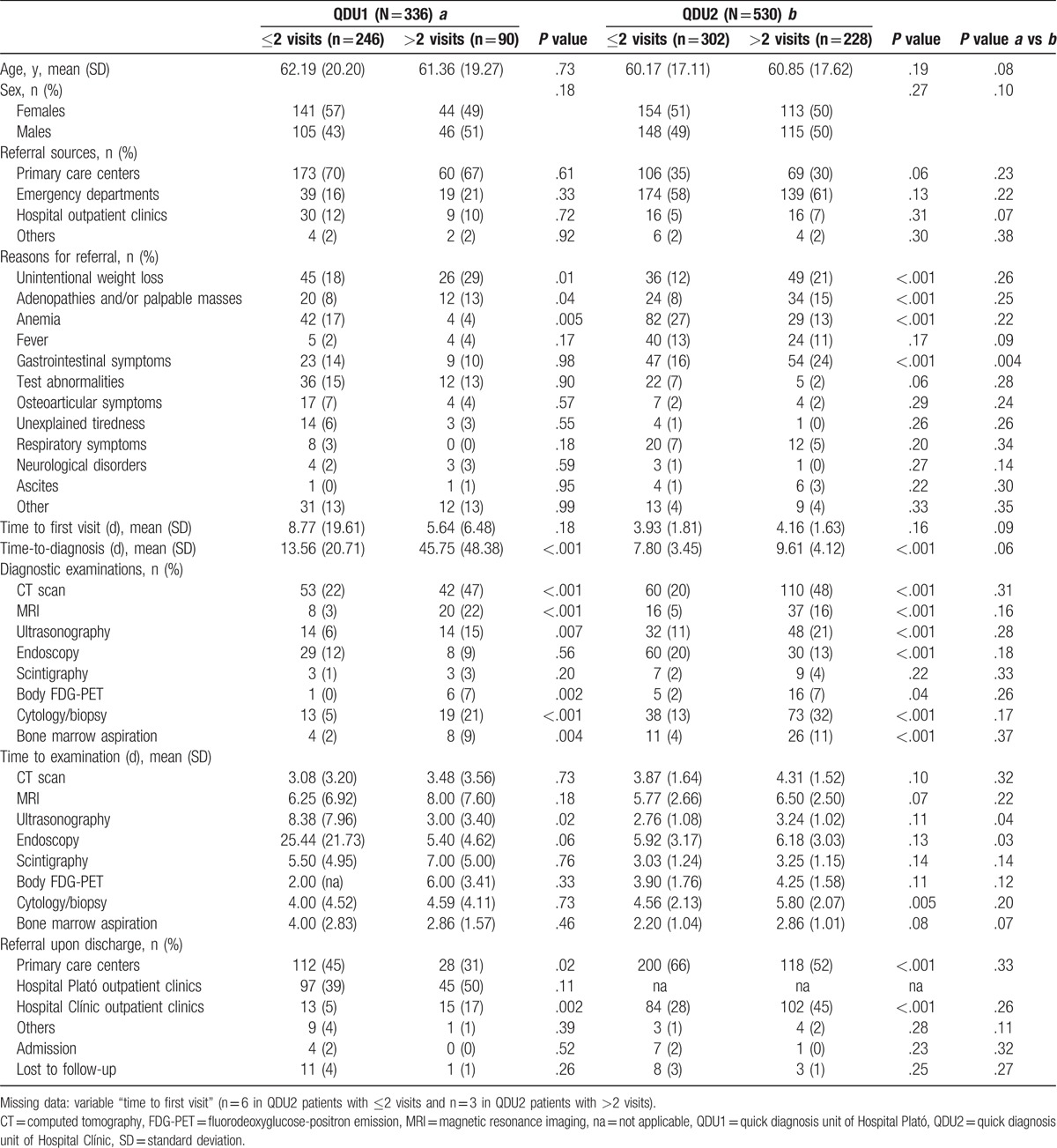
Comparative analyses according to number of visits.

**Table 5:**
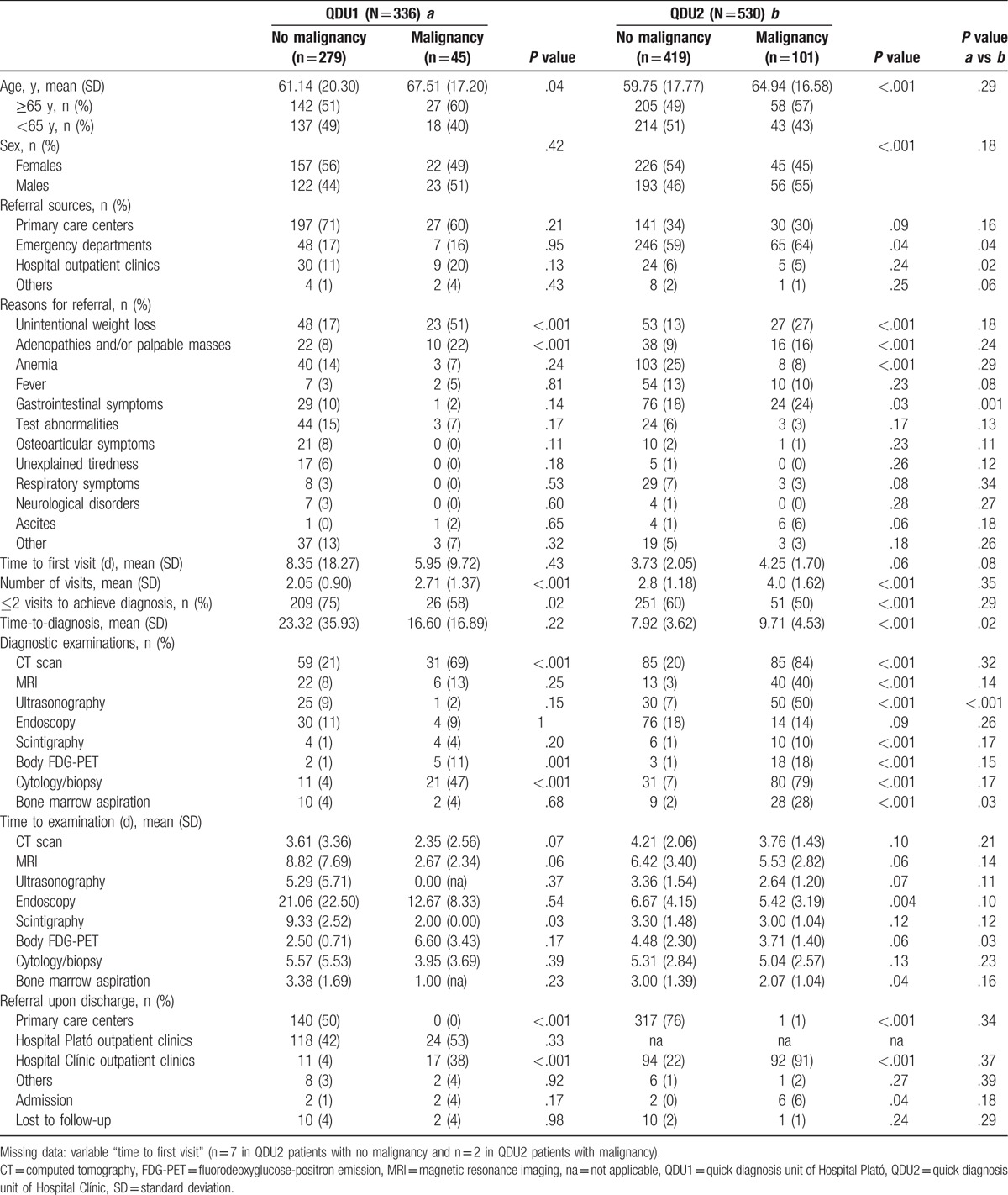
Comparative analyses according to final diagnosis.

Analysis according to the number of visits needed to make a final diagnosis revealed that QDU1 patients with >2 visits were more likely than those with ≤2 visits to have UWL and adenopathies and/or palpable masses but less likely to have anemia as referral reasons. While time-to-diagnosis was longer in QDU1 patients needing >2 visits, these patients more commonly underwent examinations than those needing ≤2 visits. Endoscopy, however, was more frequently conducted in the latter yet without statistical significance. Moreover, QDU1 patients with >2 visits were more often referred after diagnosis to the reference hospital specialist outpatient clinics. Similar results were observed in QDU2 patients: patients needing >2 visits were more likely than those needing ≤2 visits to have UWL, adenopathies, and/or palpable masses but less likely to have anemia as referral reasons. Unlike QDU1 patients, however, gastrointestinal symptoms were more frequent among QDU2 patients with >2 visits. Also, similar to the QDU1 group, QDU2 patients needing >2 visits had a longer time-to-diagnosis, underwent examinations more frequently than those needing ≤2 visits with the exception of endoscopy, which was more often performed in the latter, and were more commonly referred onward to the hospital outpatient clinics. Comparative analysis of the 2 groups according to the number of visits revealed significant differences in gastrointestinal symptoms as referral reason and in the waiting times to ultrasonography and endoscopy (Table 4).

Lastly, the most remarkable significant differences according to the final diagnosis are shown in Table 5. In particular, QDU1 patients with malignancy were older than those without it and were more likely to have UWL and adenopathies and/or palpable masses, to have a higher mean number of visits, to need less often ≤2 visits, to undergo more CT scans, body FDG-PETs and cytology/biopsy studies, and to be referred onward to outpatient clinics of the reference hospital. A similar pattern was seen in QDU2 patients: patients with cancer were older and were more likely to have UWL and adenopathies and/or palpable masses as referral reasons. Although anemia was less frequent in both QDU2 and 1 patient with cancer, statistical significance was not reached in the latter. In addition, gastrointestinal symptoms as referral reason were more common in QDU2 patients with cancer. QDU2 patients with cancer were also more likely than those without it to be males, to be initially referred from the ED, and to undergo more examinations except endoscopy, which was more commonly conducted, albeit without statistical significance, in both QDU2 and 1 patient without cancer. Unlike QDU1 patients, however, time-to-diagnosis was longer in QDU2 patients with malignancy than in those without it. Moreover, QDU2 patients with cancer were more commonly hospitalized and were more often referred onward to the hospital outpatient clinics, with only 1% being referred to PCCs. When comparing the 2 QDU groups according to final diagnosis, statistically significant differences were observed in the sources of referral, gastrointestinal symptoms as referral reason, time-to-diagnosis, number of patients undergoing ultrasonography and bone marrow aspiration, and in the waiting time to body FDG-PET (Table 5).

## 4 Discussion

To the best of our knowledge, this is the first study to compare the performance of 2 hospital-based QDUs. Despite some differences, our results show that, for diagnostic purposes, the overall performance and effectiveness of QDUs of urban district and reference general hospitals in evaluating patients with predefined referral criteria and suspected serious conditions were similar.

Among the differences observed, the ED clearly surpassed PCCs as referral source in QDU2. This may partly indicate that, in contrast to PCCs, patients presenting to the ED of the reference hospital can be directly referred to QDU2 without previous checking by the physician in charge of this unit.^[24,28]^ Although PCC physicians are aware of the referral criteria to QDU2, some patients do not meet these criteria and are instead referred to other units as decided by the QDU2 physician when assessing the PCC referral reports.^[28]^ Considering the amount of PCC referral reports to QDU2, without previous checking, the number of patients referred to the unit would surpass the number referred from the ED (data not shown). Although the original referral sources of patients to the 2 units undoubtedly determine the clinical reasons for referral,^[24,28]^ the main causes did not differ substantially, with some exceptions. In general, QDU2 patients presented with more “urgent” conditions such as anemia (mostly corresponding to severe microcytic anemia requiring transfusion),^[29,30]^ fever^[27]^ and gastrointestinal symptoms including rectorrhagia, dysphagia, and unexplained severe abdominal pain (data not shown) (Table 1). Conversely, QDU1 patients had a higher frequency of less “urgent” or “severe” disorders than QDU2 patients such as UWL, test abnormalities, osteoarticular symptoms, and unexplained tiredness. These differences most likely reflected the differences in the referral sources (Table 1).

A salient difference was observed in the time to first visit, which was quicker in QDU2 than in QDU1. This could be explained both by the direct appointments to QDU2 at ED and the administrative support of the unit.^[24,28]^ Furthermore, although patients evaluated in QDU1 required less visits to be diagnosed, the time-to-diagnosis was significantly longer than in QDU2, a finding related to the unavailability of administrative staff in the former (see below). Although the frequency of cancer as a final diagnosis in the 2 units is consistent with former reports,^[21,22,24,26,31]^ its significantly higher occurrence in QDU2 than in QDU1 patients is mostly justified by the considerably higher rate of digestive malignancies (mainly colorectal and pancreatic cancer) among patients presenting with gastrointestinal symptoms (24% in QDU2 vs 2% in QDU1) (data not shown).

It is of note that QDU activity and reasons for referral according to patients’ age, number of visits, and final diagnosis have not been analyzed previously. Patients from the 2 groups with cancer shared several characteristics: they were older and more often (*P* not significant in QDU1) males than those without cancer, had more commonly UWL and adenopathies and/or palpable masses but less commonly (nonsignificantly in QDU1) anemia, and underwent more examinations except endoscopies, which were less frequently conducted, yet without significance, in patients from the 2 groups. However, while time-to-diagnosis was nonsignificantly shorter in QDU1 patients with cancer than in those without it, it was significantly longer in QDU2 patients with cancer. The longer waiting times to some diagnostic tests in QDU2 compared with QDU1 patients with cancer explicate this difference (e.g., 5.04 vs 3.95 days to cytology/biopsy studies, respectively, or 3.76 vs 2.35 days to CT scan, respectively) (Table 5). With regard to QDU activity and referral reasons according to patients’ age, the 2 groups also shared some features: patients aged ≥65 years were more likely than younger patients to be males (nonsignificantly in QDU1), to have (nonsignificantly in QDU1) UWL and adenopathies and/or palpable masses but less likely (nonsignificantly in QDU2) fever, and to be referred onward to outpatient clinics (nonsignificantly in QDU1). A similar pattern was observed when considering the QDU performance and reasons for referral according to the number of visits. Briefly, similar to patients with a diagnosis of malignancy, patients needing >2 visits were more often, although nonsignificantly, males, had more commonly UWL and adenopathies and/or palpable masses but less commonly anemia as referral reasons, and underwent more examinations than those needing ≤2 visits except endoscopies, which were more frequently conducted in the latter (nonsignificantly in QDU1). Also, patients with >2 visits were more often referred onward to outpatient clinics, while those with ≤2 visits were more commonly referred to PCCs.

Although several characteristics of the functioning and outcomes of QDU1 and the patients evaluated at it are not substantially different from those reported in other QDUs,^[19,24,26,31]^ a key difference lies in the resources the unit at the district hospital has. While the unit of the reference hospital has an attending physician full-time and administrative and nursing staff to arrange appointments and deliver healthcare nursing work, respectively,^[24]^ QDU1 has an attending physician part-time but neither administrative nor nursing staff of its own. Nevertheless, whereas the amount of patients evaluated at the reference center unit is considerably greater and justifies a full team of healthcare and administrative professionals, an unreported pilot study concluded that the number of patients evaluated at QDU1 was proportional to the part-time work of the physician in charge of it. Yet, the unavailability of administrative personnel poses obvious organization problems for several actions, most notably appointments, with potential negative consequences in achieving, among other things, proper timings including quicker times from referral to first visit and quicker times to diagnosis. It is of note that the QDU1 attending physician herself undertakes most of the administrative work.

Comparing the QDU of a urban district hospital with the unit of a general hospital, which is in addition the reference center of the former, yielded some interesting findings. Because the former is a small hospital with a smaller reference population, some diagnostic tests ordered by the QDU physician (e.g., CT scans and cytology/biopsy studies (Table 2)) were performed in fairly short times—even faster than the same tests ordered by the physician in charge of the QDU of the reference hospital. A similar observation was reported in the QDU of the smaller community Hospital of Granollers.^[26]^ The pattern of onward referrals is also a distinctive feature. Unlike tertiary hospitals with their outpatient clinics run by physicians from a broad range of specialties, smaller hospitals commonly refer patients with specific conditions other than prevalent chronic diseases to those specialists at reference centers, predominantly for treatment purposes. Indeed, a significant number of patients diagnosed with serious conditions at QDU1 (e.g., 38% with cancer) were referred onward to specialist outpatient clinics of the reference hospital, mainly oncology clinics (Table 5).

### 4.1 Limitations

While the design of the study allowed us to analyze homogeneous variables including clinical and QDU variables, it should be read in the context of its limitations. First, despite the prospective assessment of QDU1 patients, the sample size was relatively small. Second, some relevant data from the QDU2 group might not have been fully captured—an inherent limitation linked to its retrospective analysis. The exclusion of QDU1 and 2 patients lost to follow-up together with QDU2 missing data could potentially introduce bias to the results and subsequent conclusions. Nevertheless, since only 23 patients were lost to follow-up and there were minimal missing data on the variable “time to first visit” the results were unlikely to be affected. Third, the handling of patients referred to hospital-based outpatient clinics such as those reported here or hospitalized for evaluating similar conditions can be different in other clinical settings, a situation relying on factors such as the institution traditions, the available resources, or the type of center. Consequently, the findings and potential implications of this study cannot be generalized. Last, a matched-pair binary logistic regression model where QDU1 cases were matched for age, sex, and time period with QDU2 cases at a ratio of 1:2 and “differences in diagnosis” were taken as the dependent variable (1 = malignancy; 0 = no malignancy) for each matched pair against the predictors of “reasons for referrals” and “diagnostic examinations” would likely provide a more powerful measure of the effectiveness of QDU1 and 2. Although matching was indeed done, the resulting sample was too small to perform a logistic analysis (data not shown).

## 5 Conclusion and implications

Although studies on QDUs are scarce, these units have proliferated exponentially both in general tertiary and smaller community hospitals, predominantly in Spain, over the last 15 years. Reported studies include descriptive, single-center studies^[26,31,32]^ and studies comparing QDUs with traditional admission for workup.^[22–27]^ Overall, results from these studies conclude that the 2 settings are similarly operative in reaching a timely diagnosis but that QDU savings from hospitalization are striking. Our study adds evidence to the convenience of QDUs in urban district hospital centers for diagnostic purposes and shows that the similarities of the unit to the unit of the general hospital outweigh the differences between them. Taken together, previous and current data could lead to a thoughtful project intended to create guidelines for a standardized infrastructure and functioning of QDUs, most likely adjusted to the type of hospital and its resources. While an economic evaluation of QDU1 and how it compares to QDU2 should be explored next, the favorable economic impact of these units with the cost-avoidance of hospitalization may encourage the establishment of uniformly operative QDUs nationwide to ultimately improve their efficiency as well as the efficiency of the national health service.
